# Robotic microsurgery for pediatric peripheral nerve surgery

**DOI:** 10.1007/s11701-024-02140-0

**Published:** 2024-10-29

**Authors:** Benedikt Schäfer, Gerrit Freund, Jörg Bahm, Justus P. Beier

**Affiliations:** 1 Division for Plexus Surgery, Department of Plastic Surgery, Hand Surgery–Burn Center, University Hospital RWTH Aachen, Pauwelsstraße 30, 52074 Aachen, Germany; 2 Department of Plastic Surgery, Hand Surgery–Burn Center, University Hospital RWTH Aachen, Pauwelsstraße 30, 52074 Aachen, Germany

**Keywords:** Peripheral nerve surgery, Pediatric nerve surgery, Nerve transfer, Robotic microsurgery

## Abstract

The technology of microsurgical robotic systems has shown potential benefit during the last decade for a variety of microsurgical procedures, such as vascular anastomoses, lymphatic anastomoses or nerve coaptation. At the same time, peripheral nerve surgery has produced ever more sophisticated nerve transfers in which the smallest nerve structures are connected to each other. Following obstetric brachial plexus injuries, nerve reconstruction surgery is often required in the first few years of life in order to improve the function of the affected arm, including nerve transfers to denervated muscles, which enable reinnervation of target muscles. In pediatric patients, these donor-nerve structures are even smaller than in adults, which further increases the demands to the microsurgeon. In this publication, we show the possible applications, capabilities and limitations of a dedicated microsurgical robotic system for nerve transfers in pediatric patients.

## Introduction

Obstetric brachial plexus palsy (OBPP) is a traction injury to the cervical nerve plexus that occurs during childbirth. With 0.38–1.56 cases per 1000 births [1], it is a rare but very serious complication of difficult births. It can result in a complex clinical situation that causes pronounced functional limitations up to complete paralysis of the upper limb. Very severe injuries with root avulsions and complete paresis of the hand are an indication for early surgical intervention. In the case of partial upper lesions the further regeneration is awaited first. If there is insufficient regeneration of the proximal muscles in the shoulder region and biceps, surgery should be indicated. A special case is the upper lesion with injury to the suprascapular nerve. This is the first branch from the superior trunk and innervates the supraspinatus and infraspinatus muscles. Especially infraspinatus muscle is important for external rotation of the shoulder joint. Shoulder development during growth is a central aspect, so muscular balance is very important. Selective reinnervation of the suprascapular nerve should be performed in order to restore the balance of muscular guidance of the shoulder joint. This can be performed via a dorsal approach close to the scapula [2]. The branch of the spinal accessory nerve (SAN) to the vertical part of the trapezius muscle is selectively transferred to the suprascapular nerve. Flexion of the elbow joint can be improved by a local nerve transfer by the so-called Oberlin transfer, where the biceps brachii and brachialis muscles, both of which are innervated by the musculocutaneous nerve, are selectively reinnervated by fascicles of the ulnar and median nerves [3]. These methods for restoring external rotation and elbow flexion, respectively, represent two very important techniques in brachial plexus and peripheral nerve surgery.

Robotic microsurgery has been continuously optimized in recent years, making it increasingly important in the field of reconstructive microsurgery. Higher precision and elimination of human tremor increases the safety of microsurgical sutures.

An important field of robotic microsurgery is surgery of the lymphatic system. Lymphovenous anastomoses in particular are a domain of supermicrosurgery, where robotic systems can greatly benefit [4–7]. Use of dedicated robotic microsurgery systems has also been increasingly advocated for the anastomosis of free flaps in breast and extremity reconstruction [8–11]. So far there are only a few individual reports for its use in peripheral nerve surgery [10, 12, 13].

Pediatric patient’s anatomy and the particular small size of structures might therefore pose an ideal future applications of robotic microsurgery. To date, there are no publications on the use of robotic microsurgery in pediatric patients, while the use of surgical robotic technology (Da-Vinci^®^ or Senhance^®^ system) in pediatric patients has already been described for example in urology [14], visceral surgery [15, 16] or otolaryngology [17].

## Material and methods

**Fig. 1:**
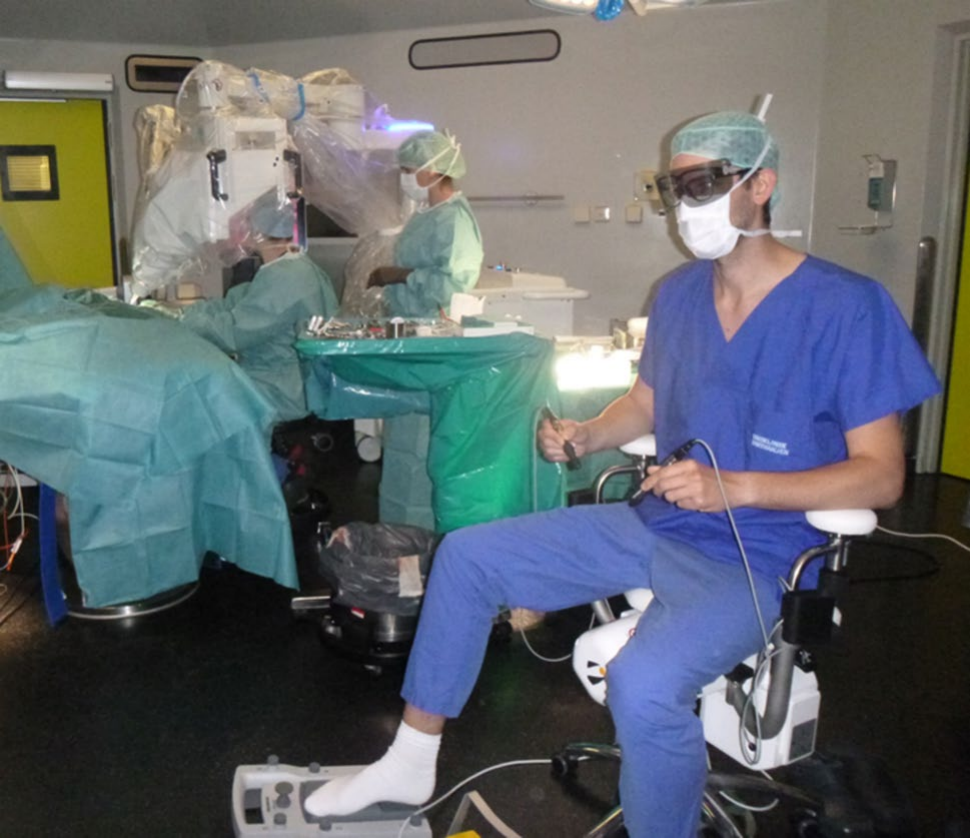
Intraoperative set up. The surgeon sits at the console and operates the robot arms using the two handles. The assistant sits sterilely next to the patient and supports the surgeon. Special glasses must be worn for the three-dimensional image using the exoscope

The dedicated microsurgical system used is the Symani Surgical System^®^ (Medical Microinstruments, MMI^®^ S.p.A, Calci, Pisa, Italy). This system can be used to work very precisely in a range of less than 1 mm. The system succeeds in completely eliminating human tremor and enables motion scaling by 7-20x. This enables a high translation of humane movements and can increase the precision and safety of the stitches. In combination we use an exoscope (ORBEYE 4 K 3D Orbital Camera System, Olympus Europa SE and Co. KG, Hamburg, Germany), which offers a maximum magnification of 26 × (13 × optical magnification combined with 2 × digital). This leaves enough space for the robot arms to be used during coaptation. The arms of the robot, to which the two instruments, i. e. microforceps and a needle holder with integrated scissors are attached, enable movements facilitated by seven degrees of freedom that are impossible for the human hand. The microsurgeon sits at a console distant to the patient, from where he operates the robotic arms (Fig. 1). The combination of a three-dimensional microscopic view with very high magnification and very precise movement of the robotic arms enables so far unrivaled accurate coaptation of these nerves. In some preliminary work, the authors describe a significantly increased comfort due to the sitting position.

**Fig. 2:**
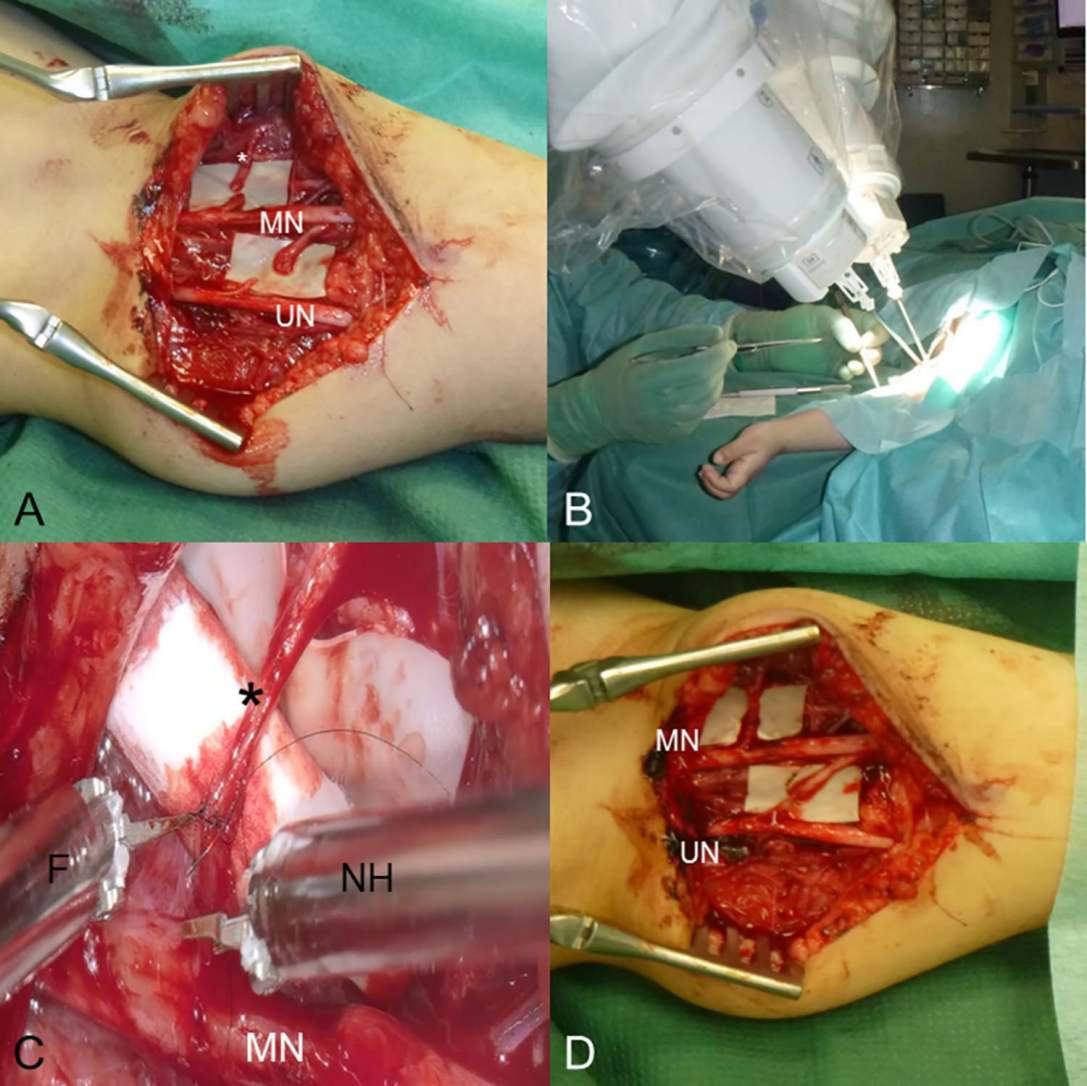
**A** Illustration of the surgical site on the left medial upper arm in a 24-month-old patient with preparation for a double fascicle nerve transfer (*UN* ulnar nerve). **B** The instruments are attached to the robot arms and allow seven degrees of freedom. **C** Magnification through the exoscope of the nerve coaptation site between a fascicle of the median nerve (MN) and the branch of the musculocutaneous nerve to the biceps brachii muscle (*). **D** Macroscopic overview after double-fascicle nerve transfer (fascicle from the ulnar nerve to the brachial muscle and fascicle from the median nerve to the biceps brachii muscle)

**Fig. 3:**
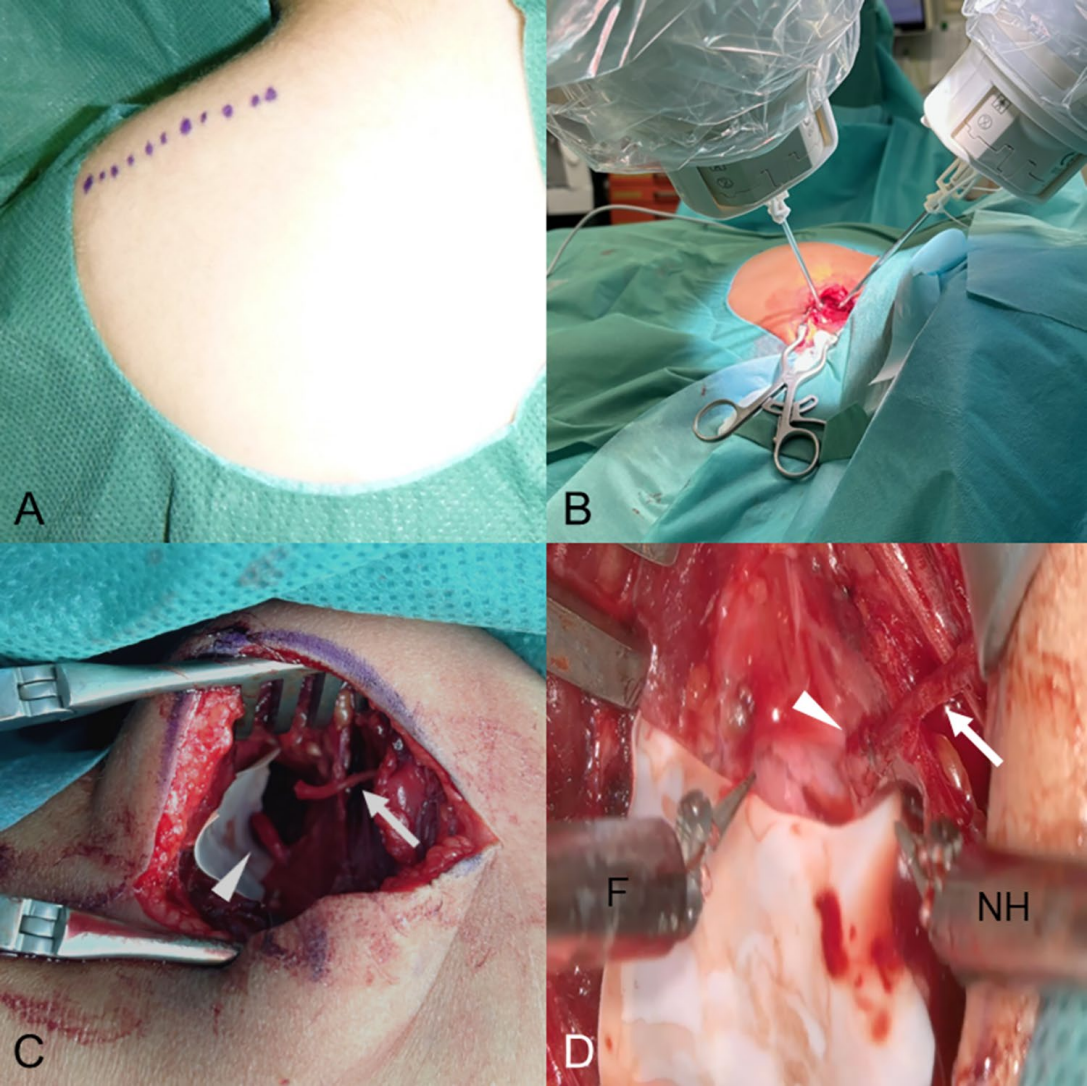
**A** Dorsal approach for the spinal accessory to suprascapular nerve transfer. Planned incision above the scapular spine. **B** Placement of the robot arms for nerve transfer. **C** Overview of the dorsal approach to the suprascapular nerve ($\triangleright$) and the SAN (→) in a 16-month-old patient. **D** Robotic micro-surgical coaptation of the SAN to suprascapular nerve. (*F* forceps, *NH* needle holder)

Informed consent was obtained by the parents for all cases. Nerve transfers were performed in patients in the age of 16–24 months to reinnervate the elbow flexor muscles, brachialis muscle and biceps brachii muscle, using double fascicle nerve transfer (Fig. 2), and to improve external rotation of the shoulder. For this purpose, half of SAN was transferred to the suprascapular nerve (Fig. 3). The transfer of the SAN was the first surgery in context of almost complete spontaneous regeneration after OBPP. The double fascicle nerve transfer was performed as a secondary procedure after previous intraplexic nerve reconstruction. Sutures were performed using Ethilon 10–0 (Ethicon, J and J, New Jersey, USA).

## Results

**Fig. 4:**
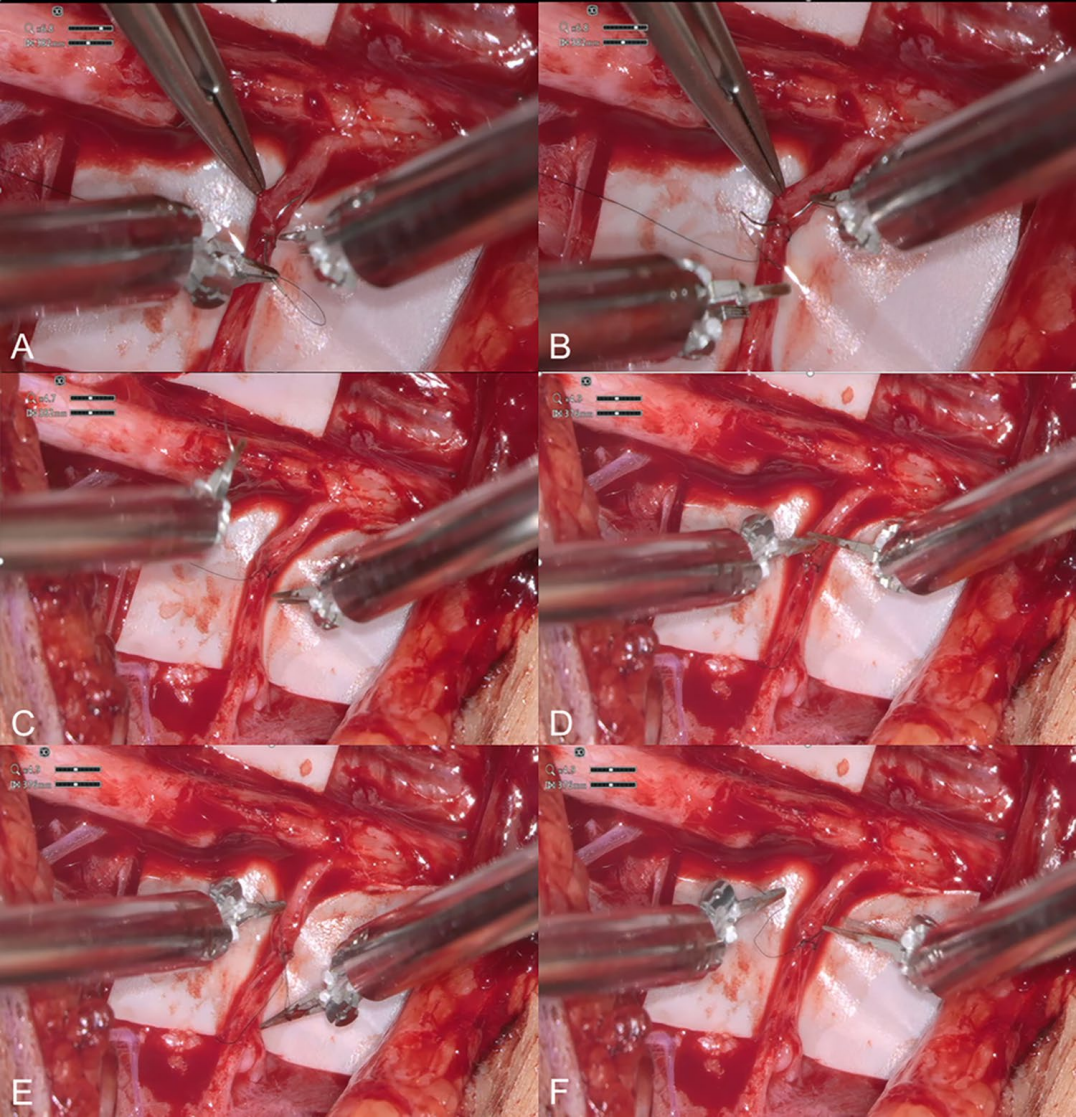
Procedure of robot-assisted microsurgical nerve coaptation. **A** Initiation of epineural nerve coaptation. Assistance from the surgical assistant with microsurgical forceps. **B** After stitching both epineuria, takeover of the needle with the forceps. **C** Pulling the suture. **D** Beginning of the knot by turning the suture around the forceps. **E** Grabbing the suture’s end. **F** Tighten the knot and cut it off with the integrated scissors on the needle holder

**Fig. 5:**
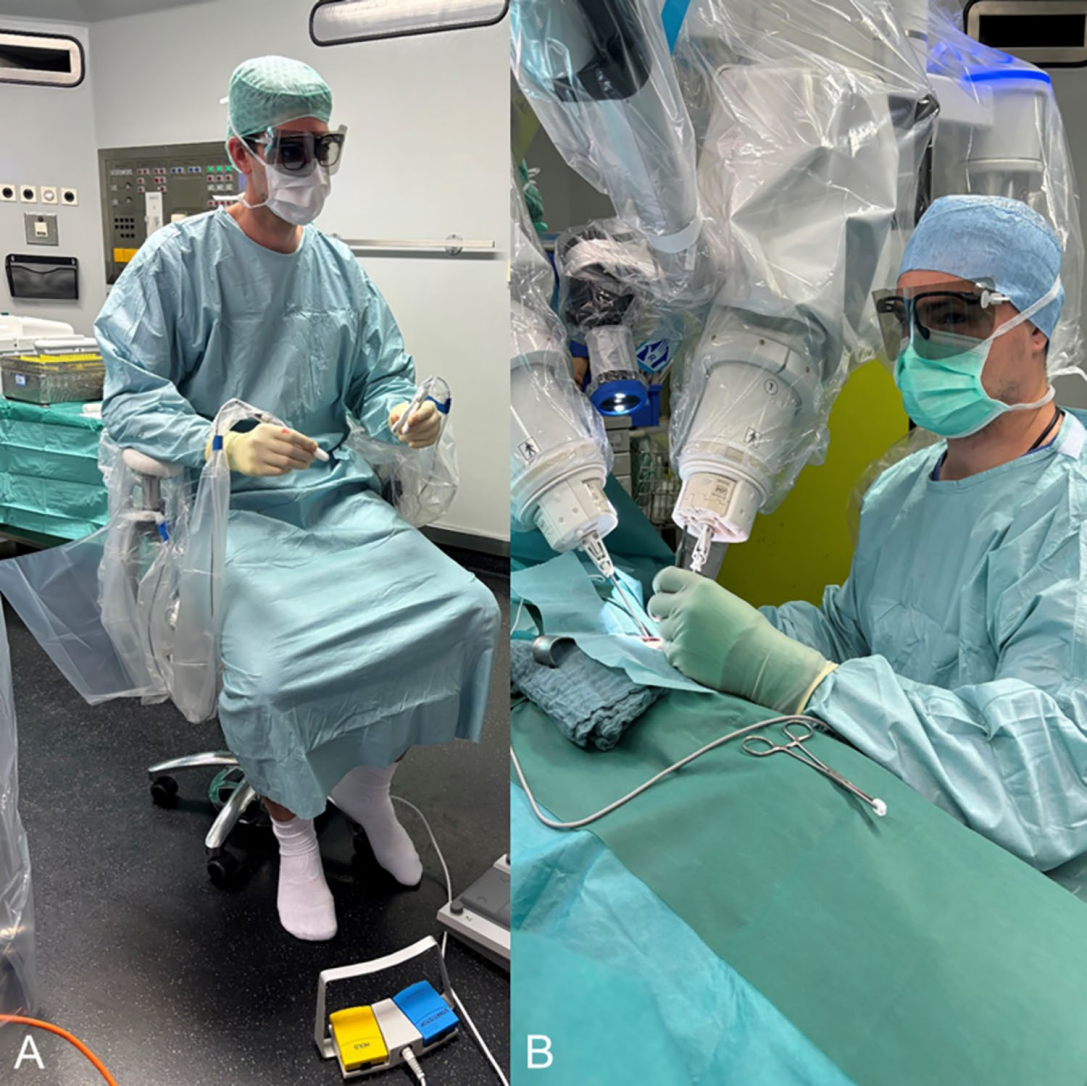
**A** The surgeon can sit at the console and move the robot arms in either sterile or non-sterile conditions (see Fig. 1). **B** The assistant has an own monitor and can support the surgeon with conventional microsurgical instruments

The surgical approach and view of the surgical site could be significantly improved by combining the Symani^®^ system and the 3D-exoscope. The system makes it easier to access hard-to-reach areas, as is particularly evident when transferring SAN to the suprascapular nerve. The 3D display in high-resolution with adequate magnification, in combination with scaling of the movement and removal of the natural tremor by the microsurgical robotic system, enables very exact and precise coaptation of the smallest nerve parts (Fig. 4). We found the comfort and improved ergonomic seating position of the microsurgeon to be advantageous (Fig. 5).

A statement on the final clinical outcome, as assessed by the range of motion of the patients, cannot be made yet, because the reinnervation period is not yet completed. The system has not yet been used for a sufficiently long period of time for this type of operation, the patients have not yet been followed up long enough to make adequate statements about the extent of movement achieved. Especially with regard to the important aspect of nerve transfer to the suprascapular nerve, a prevention of glenohumeral dysplasia, patients must be followed up for several years. However, based on our many years of experience with exactly these nerve transfers and the observations of many patients, we expect correspondingly good results, which we know from patients treated with conventional microsurgery. The nerve transfer techniques per se, i. e. the indication, the choice of donor nerve and the coaptation itself, ultimately remains unchanged by the new method shown here. We therefore expect the results to be as described in the literature.

The Oberlin procedure as a safe method to restore elbow flexion in children with OBPP was described by de Matos Figueiredo et al. They showed that after one year all treated children reached at least the mouth with the hand without any loss of function in wrist flexion [18]. With a follow-up period of 19 months, Siquera et al. came to the same conclusion that the Oberlin procedure safely and reliably delivers good to very good results, with strength of M3-M4 (Medical Research Council/MRC) [19]. These reliable results are consistent with those described by other authors as well as based on our own clinical experience [20, 21].

Improving external rotation by transferring the SAN to the suprascapular nerve is a frequently used procedure. For the average reanimation of the external rotation about 9–15 months are described until a sufficient assessment of the function is possible [22]. In average, results can be achieved in which approx. 30° external rotation is described from the neutral position [23]. In many cases a glenohumeral dysplasia can be successfully prevented by this nerve transfer [24]. We also recognize the results described in our everyday clinical work. Nevertheless, the described nerve transfers do not ultimately succeed with absolute certainty. In particular, the balance of the shoulder muscles and the improvement of external rotation must in some cases be achieved through muscle transfers.

## Discussion

With the application possibilities presented here, we were able to demonstrate that the safety and precision of nerve coaptations in pediatric patients during reconstruction following OBPP are ensured. Exact coaptations are of great importance in peripheral nerve surgery so that as many axons as possible can sprout correctly and as little fibrosis or neuroma as possible develops. This has enabled us to expand the applications of the microsurgical robotic system and also add a valuable option in the field of peripheral nerve surgery in pediatric patients. Since we first introduced the application of the microsurgical robotic system in peripheral nerve surgery [12], few further descriptions of the application of the Symani^®^ system in peripheral nerve surgery have been published [10, 25]. However, no publication has addressed the application of a dedicated microsurgical robotic system for peripheral nerve surgery in pediatric patients.

Naito et al. presented the implementation of an Oberlin procedure using the da Vinci robotic system [26] in three adult patients. They performed an open approach to the nerves in three cases, in one case they attempted an endoscopic, minimal invasive approach to the nerves. However, this did not work, because CO_2_ insufflation was not adequately successful. The nerve coaptations were performed using the robotic system. However, the advantage of the minimally invasive approach using the Da Vinci system is very unreliable in peripheral nerve surgery and in many cases were converted to the conventional approach. Moreover, the instruments of the system are not specialized for microsurgical use [27]. A very recent review of the use of robotic surgery in pediatric patients showed no application of peripheral nerve surgery [28]. This shows that previous systems could not be used effectively in this field. In our opinion, the Symani^®^ system achieves this possibility. In particular, the possibility of movements that are not possible for human’s hand makes nerve coaptation in hard-to-reach areas easier [29]. The seven degrees of freedom of the robot arms in particular make movements possible that the human hand cannot perform. In addition, the combination with an 3D-exoscope can provide a view of the surgical site that is not possible with a conventional surgical microscope and standard instruments. An important point of comfort is that the console can be used both sterile and non-sterile. This gives the surgeron the opportunity to choose and be more flexible.

As one drawback, the Symani^®^ microrobotic system can currently only be used for microsurgical suturing due to the lack of a microsurgical robotic scissor. The dissection and preparation of such delicate structures as pediatric nerve fascicles is therefore only possible to a very limited extent or not at all with such microrobotic systems, but in future development of further microsurgical instruments can be expected. Furthermore, the use of a microsurgical robot system comes along with a learning curve for the microsurgeon. Intensive training with the system is a prerequisite for using it on patients, but after a certain learning period, duration for anastomotic procedures soon level up with conventional microsurgical anastomoses/coaptations [30]. Other authors were able to observe a good learning curve for vascular anastomoses [31]. Regarding nerve copatations no significant change in the time per stitch could be observed [25]. At the moment, application of the Symani^®^ robotic system still causes high additional costs which hopefully will be overcome with broader application of such systems in future.

## Conclusion

The complete reduction of human tremor and the up to 20 × motion scaling of the microsurgical robotic system enables very precise coaptations of the smallest nerve structures in pediatric patients. In this manuscript, we also show that the microsurgical robotic system offers great advantages when accessing structures that are difficult to reach, particularly in the case of nerve transfer of the SAN to the suprascapular nerve. Despite the economic burden and the need for initial intensive training by the microsurgeon, the described advantages of the system outweigh those disadvantages, holding promise for broader application in future pediatric nerve surgery. We will certainly strive for a long-term evaluation of our patients, including a comparison with robotic microsurgery, in future.

## Data Availability

No datasets were generated or analysed during the current study.
